# Supplementary material for: CLASPP: A unified model for predicting post-translational modifications
Source: PLoS Comput Biol. 2026 Aug 14;22(8):e1014616. doi: 10.1371/journal.pcbi.1014616 (PMC13475974; doi:10.1371/journal.pcbi.1014616)
Supplement: Supplementary file 1 [file pcbi.1014616.s007.pdf]

The paper introduces **CLASPP**, a novel, unified deep learning model designed to overcome key limitations in predicting multiple Post-Translational Modifications (PTMs) from protein sequence data. It addresses the significant challenge of data imbalance across various PTM types by employing unsupervised clustering-based undersampling and a hierarchical data curation process. Built upon an ESM-2-150m encoder, the model is trained in a three-stage regimen that incorporates masked language modeling, a novel supervised contrastive learning framework tailored for PTM data, and a final fine-tuning step.

Comment 1:
A comprehensive description of the dataset size is required. Please provide detailed information on the number of samples for each PTM type at every stage of the workflow — including before clustering, after clustering, during contrastive learning, during classification head training, and during inference. Clear quantification at each stage will enhance transparency and reproducibility.

Comment 2:
A detailed explanation of the 54 binary labels is needed. In particular, the negative labels appear dependent on the corresponding positive labels (i.e., a site is typically considered negative when the predicted probability for the positive label is low). Please clarify the rationale for explicitly defining negative labels in this context.

Additionally, labels were defined based on the number of sub-clusters rather than PTM types. This labeling strategy could introduce interpretational ambiguity — for instance, a site might be predicted as positive for both phospho cluster 1 and phospho cluster 2. Please justify the adoption of this labeling scheme despite the potential overlap it introduces.

Comment 3:
Please specify which feature representations were used for spectral clustering. It would be helpful to clarify whether one-hot encoding, ESM embeddings, or alternative representations served as the feature inputs.

Comment 4:
A clearer justification for the choice of K(number of clusters) is needed. As mentioned around line 192, evaluating performance by training individual classification models for each sub-cluster may not constitute sufficient evidence, since intra-cluster similarity could naturally yield inflated performance. Please consider including additional validation metrics or visualizations to more robustly support the selected K value.

Comment 5:
Please justify the use of a 21-residue window for model input. Supporting evidence — such as comparisons with different window sizes, references to prior studies, or biological reasoning related to known PTM site context — would strengthen this design choice.

Comment 6:
Figure 3C presents results only for phosphorylation. It would be informative to assess whether similar embedding separation patterns are observed for other PTM types, either across categories or among sub-clusters within a single PTM type. Such comparisons could reveal whether the observed contrastive learning effects generalize beyond phosphorylation.

Comment 7:
We compared the CLASPP model predictions using sites annotated in our in-house dbPTM dataset (numbers in parentheses indicate sample counts) and observed discrepancies relative to Figure 4. To ensure consistent and reliable evaluation, it would be beneficial to establish a clearly defined benchmark dataset. In addition, please consider presenting performance metrics separately for each PTM type to facilitate more interpretable

comparisons.

	Acc	Precision	Recall	F1-score	AUROC	AUPRC
S_Phosphorylation (26,618)	0.5305	0.5449	0.3538	0.429	0.5493	0.5342
T_Phosphorylation (10,279)	0.5121	0.5745	0.122	0.2013	0.5518	0.5442
Y_Phosphorylation (4,697)	0.5014	0.5002	0.9744	0.6611	0.5156	0.5146
K-Acetylation (6,790)	0.5303	0.5592	0.2785	0.3719	0.5623	0.5456
K-Methylation (315)	0.5587	1	0.0974	0.1775	0.6855	0.704
R-Methylation (607)	0.5288	0.5163	0.9869	0.6779	0.609	0.6322
C-Glutathionylation (168)	0.6429	0.6053	0.8214	0.697	0.7279	0.7456
P-Hydroxylation (145)	0.4552	0.4783	0.5867	0.5269	0.4458	0.4952
	Accuracy	F1	Precision	Recall	AUC-ROC	AUC-PRC
macro metric	0.5289	0.5112	0.527	0.5241	0.5409	0.526
micro metric	0.5242	0.5242	0.5242	0.5242	0.5409	0.526

Comment 8:
It would be valuable to evaluate the model’s performance on a dataset containing multi-PTM sites, where a single residue is annotated with multiple PTM types. Please consider reporting results under the criterion that a prediction is considered correct only when all annotated PTM types at a given site are accurately identified.

Minor Comments

- Some figure captions (e.g., Fig. 2 and Fig. 3) could benefit from more descriptive explanations of abbreviations or variables.
- Providing a summary table of the 12 PTM types, their data counts, and final label mappings would enhance readability.
