# Supplementary material for: CLASPP: A unified model for predicting post-translational modifications
Source: PLoS Comput Biol. 2026 Aug 14;22(8):e1014616. doi: 10.1371/journal.pcbi.1014616 (PMC13475974; doi:10.1371/journal.pcbi.1014616)
Supplement: Supplementary file 2 [file pcbi.1014616.s008.pdf]

Review

CLASPP: A unified model for predicting post-translational modifications

This manuscript presents CLASPP, a unified deep learning model for multi-PTM prediction, built upon a sophisticated three-stage training strategy. The work addresses a significant need in the field for moving beyond single-PTM or ensemble models toward a more streamlined and powerful solution. The key innovation lies not just in the model architecture but in the thoughtful integration of stratified data curation with a supervised contrastive learning phase, which aims to learn better separated and biologically meaningful representations.

The manuscript succeeds in providing a comprehensive evaluation, demonstrating state-of-the-art performance against existing multi-PTM predictors on a carefully curated benchmark. The authors compellingly show that the contrastive learning stage enhances feature separation, and they provide evidence that these features align with known biology, such as kinase specificity profiles. The analysis of out-of-distribution performance across species and the application to an understudied kinase are particularly valuable for assessing the model's practical utility and limitations. The model's unified approach, which avoids the complexity of stitching together multiple specialist models, is a notable strength.

To further strengthen this impressive work and its potential impact, the following points are suggested for consideration:

1. The text states that CLASPP is a "streamlined, single-model solution" compared to ensemble methods. Beyond streamlining, did you demonstrate that CLASPP's performance is comparable or superior to a custom-built ensemble of single-PTM predictors on the same data?
2. What do you identify as the primary limitation of the current CLASPP model? Is it the restriction to 21-mer sequences, the specific set of PTMs covered, or something else? Could you elaborate?
3. The analysis uses kinase substrate specificity (a known biological feature) to validate the clusters, but the "enriched sub-clusters" themselves were likely *defined* by this same knowledge. This risks a circular argument where you're finding what you already decided to look for.
4. Could the observed alignment with kinase specificity be a self-fulfilling prophecy, since the sub-clusters were likely identified based on enrichment for these very motifs?"
5. Given UMAP's sensitivity to hyperparameters, can you demonstrate that the improved separation in Figure 3c is a robust result of contrastive learning and not an artifact of the visualization settings?
6. The sequence logos show *what* the model sees (amino acid preferences), but can you provide any evidence for *how* it informs kinase-specific biology beyond these known motifs?
7. The test set was 'curated from unsupervised sub-clusters.' Please detail the exact procedure to ensure this curation did not introduce data leakage, and confirm the clustering was performed *only* on a designated training portion of the data
8. How do you explain the physiologically improbable, perfect AUC_PRC scores (e.g., 1.0) for several PTMs? Does this suggest the test data for these PTMs is too easy, redundant with the training set, or potentially artifactual?
9. The DCLK3 case, while compelling, is an anecdote (N=1 protein). The fact that CLASPP and DeepMVP have high false positives in this example directly contradicts the narrative of overall superiority and highlights a key trade-off (recall vs. precision) that is glossed over.
