# Supplementary material for: CLASPP: A unified model for predicting post-translational modifications
Source: PLoS Comput Biol. 2026 Aug 14;22(8):e1014616. doi: 10.1371/journal.pcbi.1014616 (PMC13475974; doi:10.1371/journal.pcbi.1014616)
Supplement: Supplementary file 3 [file pcbi.1014616.s009.pdf]

Review 1

Overview

The paper introduces CLASPP, a novel, unified deep learning model designed to overcome key limitations in predicting multiple Post-Translational Modifications (PTMs) from protein sequence data. It addresses the significant challenge of data imbalance across various PTM types by employing unsupervised clustering-based undersampling and a hierarchical data curation process. Built upon an ESM-2-150m encoder, the model is trained in a three-stage regimen that incorporates masked language modeling, a novel supervised contrastive learning framework tailored for PTM data, and a final fine-tuning step.

Comment 1: A comprehensive description of the dataset size is required. Please provide detailed information on the number of samples for each PTM type at every stage of the workflow — including before clustering, after clustering, during contrastive learning, during classification head training, and during inference. Clear quantification at each stage will enhance transparency and reproducibility.

Response: We thank the reviewer for the insightful comments and suggestions to improve the clarity and transparency of our work. In response to the reviewer's comment, we have revised the manuscript and updated Supplementary Figure 2 (S2e Fig) to indicate the dataset size at each stage of the data curation and training workflow.

Comment 2: A detailed explanation of the 54 binary labels is needed. In particular, the negative labels appear dependent on the corresponding positive labels (i.e., a site is typically considered negative when the predicted probability for the positive label is low). Please clarify the rationale for explicitly defining negative labels in this context. Additionally, labels were defined based on the number of sub-clusters rather than PTM types. This labeling strategy could introduce interpretational ambiguity — for instance, a site might be predicted as positive for both phospho cluster 1 and phospho cluster 2. Please justify the adoption of this labeling scheme despite the potential overlap it introduces.

Response: We thank the reviewer for this question. Since Reviewer #2 requested similar clarification, we have revised the methods sections (sections titled: “Negative Data Handling” and “Model design”) to clarify our labeling strategy. The use of the negative class is to differentiate hard negatives and easy/medium negatives. We chose to include a separate negative label to allow the model to differentiate hard and easy/medium negative

data. Additionally, the sub-cluster class predictions that all belong to the same PTM are max pooled, so predictions end up being 19 separate residues specific PTM labels and 1 easy/medium label (20 in total). The reason for max pooling is to align our labeling scheme with that of previously published studies, to enable comparative benchmarking of the different models.

Comment 3: Please specify which feature representations were used for spectral clustering. It would be helpful to clarify whether one-hot encoding, ESM embeddings, or alternative representations served as the feature inputs.

Response: We agree that our description of spectral clustering and its associated input features was not initially clear. We have updated the "Unsupervised Clustering" methods section to highlight the input features for each clustering method. We end up using the 21mer peptides themselves as input and the 3D volumetric (Å^3) size per residue as inputs for the clustering techniques.

Comment 4: A clearifer justification for the choice of K(number of clusters) is needed. As mentioned, around line 192, evaluating performance by training individual classification models for each sub-cluster may not constitute sufficient evidence, since intra-cluster similarity could naturally yield inflated performance. Please consider including additional validation metrics or visualizations to more robustly support the selected K value.

Response: We thank the reviewer for suggesting an additional way to validate the number of clusters for each PTM type. We have added the mean with-cluster distance and mean silhouette coefficient to supplementary figure 2 (S2c Fig) for every k number of clusters. These cluster quality metrics generally correlate with performance.

Comment 5: Please justify the use of a 21-residue window for model input. Supporting evidence — such as comparisons with different window sizes, references to prior studies, or biological reasoning related to known PTM site context — would strengthen this design choice.

Response: We thank the reviewer for suggesting an additional hyperparameter to investigate. We have cited previous works in the newly added 4th paragraph of the discussion sections that cover varied context window size limitations and other aspects such as model size and parameter choices.

Comment 6: Figure 3C presents results only for phosphorylation. It would be informative to assess whether similar embedding separation patterns are observed for other PTM types, either across categories or among sub-clusters within a single PTM type. Such comparisons could reveal whether the observed contrastive learning effects generalize beyond phosphorylation.

Response: This comment was also provided by Reviewer #2. To address these comments, we have included additional pre and post-contrastive learning UMAP projections for all participating PTM types to a newly added S3 Figure. This additional data figure demonstrates embedding separation for most PTM types post contrastive learning for the PTM types such as phosphorylation, ubiquitination, acetylation, glycosylation, and methylation.

Comment 7: We compared the CLASPP model predictions using sites annotated in our in-house dbPTM dataset (numbers in parentheses indicate sample counts) and observed discrepancies relative to Figure 4. To ensure consistent and reliable evaluation, it would be beneficial to establish a clearly defined benchmark dataset. In addition, please consider presenting performance metrics separately for each PTM type to facilitate more interpretable comparisons.

Response: We thank the reviewer for bringing these reproducibility concerns to our attention. Since this was also raised by reviewer 3, we have significantly revamped the Zenodo repository to provide a uniform format and working link (https://zenodo.org/records/17674057) for the benchmarking dataset. In the revamped repository, we indicate the benchmark dataset used for each figure to enable reproducibility. We also include an additional supplementary figure (S4 Fig) to show PTM specific metric scores for each model.

Comment 8: It would be valuable to evaluate the model's performance on a dataset containing multi-PTM sites, where a single residue is annotated with multiple PTM types. Please consider reporting results under the criterion that a prediction is considered correct only when all annotated PTM types at a given site are accurately identified.

Response: We thank the reviewer for suggesting additional benchmarks and have added the multi-PTM site benchmark in the new S4b figure to demonstrate multi-PTM site / PTM crosstalk performance rather than averaged single PTM performance. Unfortunately, this

does not show that CLASPP has SOTA performance when compared to a metric where a single residue is annotated with multiple PTM types, presumably because of our data curation, limiting the examples of multi-PTM events to 20% of the data included. We still included the benchmark in Figure S4. and mentioned the limitations of our model in multi-PTM prediction in the results section titled "Performance evaluation and comparison to other models".

Minor Comments

Minor Comment 1: Some figure captions (e.g., Fig. 2 and Fig. 3) could benefit from more descriptive explanations of abbreviations or variables.

Response: We thank the reviewer for pointing out comments that would increase clarity. We updated figure 2 and 3 to reflect this point.

Minor Comment 2: Providing a summary table of the 12 PTM types, their data counts, and final label mappings would enhance readability

Response: We thank the reviewer for pointing out comments that would increase clarity. We have added additional supplemental S2 Fig. to include this.

Review 2

Overview

This manuscript presents CLASPP, a unified deep learning model for multi-PTM prediction, built upon a sophisticated three-stage training strategy. The work addresses a significant need in the field for moving beyond single-PTM or ensemble models toward a more streamlined and powerful solution. The key innovation lies not just in the model architecture but in the thoughtful integration of stratified data curation with a supervised contrastive learning phase, which aims to learn better separated and biologically meaningful representations. The manuscript succeeds in providing a comprehensive evaluation, demonstrating state-of-the-art performance against existing multi-PTM predictors on a carefully curated benchmark. The authors compellingly show that the contrastive learning stage enhances feature separation, and they provide evidence that these features align with known biology, such as kinase specificity profiles. The analysis of out-of-distribution performance across species and the application to an understudied kinase are particularly valuable for assessing the model's practical utility and limitations. The model's unified approach, which avoids the complexity of stitching together multiple

specialist models, is a notable strength. To further strengthen this impressive work and its potential impact, the following points are suggested for consideration:

Comment 1: The text states that CLASPP is a "streamlined, single-model solution" compared to ensemble methods. Beyond streamlining, did you demonstrate that CLASPP's performance is comparable or superior to a custom-built ensemble of single-PTM predictors on the same data?

Response: We thank the reviewers for asking about our streamlined model and potential performance improvements. Our current benchmark for Fig. 4 and S4 Fig. includes PTMGPT2, MTPrompt-PTM, DeepMVP, MIND-S, and MusiteDeep which all consist of an ensemble model framework.

Comment 2: What do you identify as the primary limitation of the current CLASPP model? Is it the restriction to 21-mer sequences, the specific set of PTMs covered, or something else? Could you elaborate?

Response: We thank the reviewer for the chance to expand upon our model's limitations. In the revised version, we have updated the discussion section to mention limitations of CLASPP model, including varied context window sizes, number of model parameters, model size, parameter freezing, and augmentation strategies.

Comment 3: The analysis uses kinase substrate specificity (a known biological feature) to validate the clusters, but the "enriched sub-clusters" themselves were likely defined by this same knowledge. This risks a circular argument where you're finding what you already decided to look for.

Response: We agree that our rational for cluster validation was not made clear in the initial version. In the revised version, we have rewritten the results section "Enhancing CLASPP with Contrastive Learning" to better reflect the motivations and the distinction between downstream analysis and what is incorporated into the curation/training. In brief, clustering was performed unsupervised, without labels or prior knowledge. Thus, any functional grouping arising from the clusters reflects true patterns in the existing data, rather than an artifact of labeling based on prior knowledge.

Comment 4: Could the observed alignment with kinase specificity be a self-fulfilling prophecy, since the subclusters were likely identified based on enrichment for these very motifs?"

Response: We thank the reviewer for pointing out the concerns in the enrichment. We have re-written the results section "Enhancing CLASPP with Contrastive Learning" to better reflect that the enrichment was only added to the analysis / visualization and not used in the curation process. The unsupervised clustering acts as an unbiased way to separate groups. Finding these motif patterns at the very least acts as quality control for the unsupervised clustering, and the UMAP projections separation act as way to understand what the model learns given that the clusters represent known biologically meaningful patterns.

Comment 5: Given UMAP's sensitivity to hyperparameters, can you demonstrate that the improved separation in Figure 3c is a robust result of contrastive learning and not an artifact of the visualization settings?

Response: To address this concern, we have performed additional experiments with different replicates, random seeds, and hyperparameters. Results from these experiments demonstrate clear differences in pre- and post-contrastive learning on all PTM types trained on (S3 Fig). These additional experiments, along with the corresponding UMAP projection, are now included as Supplementary Figure 3 in the revised version.

Comment 6: The sequence logos show what the model sees (amino acid preferences), but can you provide any evidence for how it informs kinase-specific biology beyond these known motifs?

Response: We have revised the results section ("Enhancing CLASPP with Contrastive Learning) to better correlate the PTM motifs to kinase substrate preferences and specificity in cellular signaling pathways.

Comment 7: The test set was 'curated from unsupervised sub-clusters.' Please detail the exact procedure to ensure this curation did not introduce data leakage, and confirm the clustering was performed only on a designated training portion of the data

Response: We thank the reviewer for pointing out this lack of clarification of how we ensure no data leakage among training/testing/validation. We have updated the methods section "Unsupervised Clustering" and "Uniform Sampling from the Sub-Clusters" to highlight details that ensure no data leakage. This was achieved by stratified sampling for the training/testing/validation split per sub-cluster as the last step in the curation.

Comment 8: How do you explain the physiologically improbable, perfect AUC_PRC scores (e.g., 1.0) for several PTMs? Does this suggest the test data for these PTMs is too easy, redundant with the training set, or potentially artifactual?

Response: We agree with the reviewer that for a subset of PTM types performance metrics (PRC-AUC) are suspiciously high. This is likely due the limited number of "hard" negatives for these PTM types, which might have resulted in inflated performance. In the revised version we revamped the positional mapping and the positive/negative ratio for all PTM types and re-ran the benchmarks (revised Fig 4, S4 Fig.) Increasing the re-covered number of "easy" negatives for these PTM types has resolved this issue.

Comment 9: The DCLK3 case, while compelling, is an anecdote (N=1 protein). The fact that CLASPP and DeepMVP have high false positives in this example directly contradicts the narrative of overall superiority and highlights a key trade-off (recall vs. precision) that is glossed over

Response: We thank the reviewer for pointing out this lack of clarification of false positivity and how CLASPP and DeepMVP both have higher FPR. We have revamped the 1st and 2nd paragraph in the discussion section describing the false positivity of CLASPP.

Review 3

Overview

Use of Contrastive Learning Framework Gravel et al. utilized a contrastive learning based, attention-driven stratified framework for predicting major PTMs.

Comment 1: Line 64 Correction Replace labile with dynamic.

Response: We thank the reviewer for pointing out clarification. We have updated the intro with the suggested change.

Comment 2: Choice of Embeddings (ESM2-3B vs. ESM-2-150M)

The manuscript does not justify why ESM2-3B contextualized embeddings were not used. Instead, the study relies on ESM-2-150M encoder embeddings. A clear rationale should be provided. Additionally, the dimensionality of the contextualized embeddings generated by the 150M model must be explicitly stated.

Response: We thank the reviewer for pointing out a lack of clarification in the choice of model architecture as well as the model dimensionality specifications. We updated S1a,b,c,d,e and h Fig. and the 4th paragraph in the discussion to reflect the limitations of CLASPP and denote embedding dimensions for all stages of training. The larger models introduce much larger computational costs during the contrastive learning stage of training.

Comment 3: Exclusion of MCC Evaluation Metric

MCC (Matthews Correlation Coefficient) has been consistently used in prior PTM prediction studies, particularly due to its robustness with imbalanced datasets. The manuscript should explain why MCC was not included as an evaluation metric.

Response: We thank the reviewer for pointing out another relevant metric to include in our evaluation. We have included this in Fig 4 and S4 Fig.

Comment 4: Figure and Typographical Corrections, Figure 3A: Change "Losss" to "Loss.", Line 381: Replace "Muti" with "Multi."

Response: We thank the reviewer for pointing out a grammatical error. We have updated the manuscript to correct these mistakes.

Comment 5: Construction of the Negative Dataset

The method used to construct the negative dataset is unclear. For example, if specific serine/threonine sites in a protein are annotated as phosphorylated, are the remaining non-annotated S/T sites in the same protein treated as negative? This is a standard requirement in PTM prediction. If this procedure was not followed, the manuscript cannot be considered acceptable. A detailed explanation of the negative data generation process is required.

Response: We thank the reviewer for asking about our negative data generation. Like other review comments, we have updated the methods section "Negative Data Handling" to include more in-depth negative data generation with motivations. We introduced more than one type of negative data to introduce a priority-based sample strategy to combat the disparity of positive/negative ratios among the different PTM types.

Comment 6: Feature Vector Generation via ESM-2-150M

The manuscript should clearly describe how each 21-mer sequence was converted into feature vectors using the ESM-2-150M encoder, including the embedding structure. Additionally, the application of contrastive learning to these feature vectors needs to be explained step by step.

Response: We thank the reviewer for raising the issue of lack of clarity in our training/forward pass schematic. We have updated S1 Fig and the method section of "Contrastive Learning" to provide a pictographic and formal description of the embeddings being used in the contrastive learning frameworks.

Comment 7: GitHub Repository

All PTM-related data (positive and negatives) should be provided in the following format: Protein ID | PTM Site | 21-mer Peptide | Label | PTM Type

Example:

P12KYKU | 21 | ..RSLNKSTUT.. | Negative | Phosphorylation

Currently, no such data is available in the GitHub repository. You must provide all datasets used in the analysis in this exact format to ensure reproducibility and enable further study.

Response: We thank the reviewer for bringing this to our attention. We have revamped the Zenodo repository and provided a working link (https://zenodo.org/records/17674057). In doing so, we have revamped easy/medium data mapping/handling and implemented the suggested "individual" format and also provided "matrix" formats for all training-testing-validation files. "Individual" benchmarks are also separate by PTM type and are provided for human and other species. Due to the abundance of files, easier figure mapping is found in the descriptions provided in the Zendo repo.
