# Supplementary material for: CLASPP: A unified model for predicting post-translational modifications
Source: PLoS Comput Biol. 2026 Aug 14;22(8):e1014616. doi: 10.1371/journal.pcbi.1014616 (PMC13475974; doi:10.1371/journal.pcbi.1014616)
Supplement: Supplementary file 4 [file pcbi.1014616.s010.pdf]

Point by point response to Reviewer #1 comments:

Reviewer #1: Comment 1: In Supplementary Figure 2E, several PTM types (e.g., Glutathionylation, S-palmitoylation, and Hydroxylation) show higher counts in Stage 4 compared to Stage 3. Given that Stage 4 clustering is performed on the dataset already filtered in Stage 3, the number of entries cannot increase. The authors should clarify this discrepancy or revise the figure accordingly.

Response: We thank the reviewer for pointing out this discrepancy. This was due to handling the multi-label PTM sites separately between Stages 2 and 4 of the curation pipeline for organization purposes. When updating the abundance of Stage 3 with the counts coming from the separate multi-label data pipeline, we get abundance counts that are comparable. Supplementary Figure 2E has been updated to reflect this.

Comment 2: While limiting the number of positive samples to 2,000 helps balance the dataset, it may reduce the diversity of training examples from larger clusters. It would be helpful if the authors clarify how data diversity was maintained during training - for example, whether different subsets were resampled across epochs or runs, or whether a cross-validation strategy was used - to ensure that model performance is not dependent on a fixed subset of the data.

Response: We have included a histogram of average Hamming distance across the total and sampled sub-clusters. We show how the diversity of input sequences is similar from the many sub-clusters in both sequence space and post-contrastive embeddings space by comparing full sub-cluster population with downsampled populations. In nearly all cases, the sub-sampled population overlaps nearly identically to the full population (S2f Fig.).

Comment 3: In Supplementary Figure 2C, the within-cluster distance is typically expected to decrease as the number of clusters increases, due to reduced intra-cluster variance. The authors should provide a more detailed explanation of the trends observed in this figure.

Response: We thank the reviewer for requesting an explanation on the optimal number of sub-clusters per PTM type. We cover this in a new section in the discussion covering relative number of sub-clusters to flanking sequence diversity surrounding the PTM site for different PTM types like phosphorylation, acetylation, and ubiquitination. This is found in the Discussion section (5th paragraph).

Comment 4: Since CLASPP defines three different types of negative samples, additional clarification is required regarding how the negative datasets were constructed and used for performance comparison with benchmark models (shown in Figure 4).

Response: In response to this comment and the clarification requested by Reviewer #3 regarding the negative dataset handling, we have added a section in the Methods describing the creation of the benchmarks. In short, we combine the test-specific sub-cluster positive data that applies to the PTM type and add the hard, medium, and easy negative data to share a residue type. This is found in the Methods section (Negative Data Handling).

Comment 5: While Supplementary Figure 4A presents model performance across PTM types, interpretation of these results is not entirely straightforward. Specifically, some PTM types appear to exhibit simultaneously high false positive rates and high precision, which is counterintuitive and makes it difficult to assess the model's predictive behavior. To improve transparency, the authors should include confusion matrices for these PTM types to clarify how these metrics were derived.

Response: As requested by the reviewer, we have included the confusion matrices for all models and PTM-specific benchmarks (S2e Fig.).

Comment 6: The authors are encouraged to report the total number of trainable parameters, as this information would help readers better assess model complexity and enable fair comparison with existing methods.

Response: We have added layer-specific dimensions in Supplementary Figure 1d and e. We have additionally added the total number of parameters for the model in the Results section (Contrastively Learned Attention-based Stratified PTM Predictor (CLASPP) model design and training).

Point by point response to Reviewer #3 comments:

Reviewer #3: Upon reviewing the negative dataset in benchmark_test_HUMAN_hd3_Acetylation(AM)-25_05_11.csv, it is clear that the current methodology deviates from the rigorous standards required for PTM prediction. The current sampling (using Hamming distance and random selection) does not reflect biological reality. In a rigorous PTM study, if a protein contains 10 Lysine (K) residues and only two (e.g., K24 and K160) are annotated as positive, the remaining eight K residues must be treated as the negative data. This is the only way to ensure the model learns to distinguish a true modification site from a biologically similar but unmodified site.

Response: We thank the reviewer for requesting additional validation on more traditional negative data handling and will include MIND-S testing benchmarks due to their use of more traditional negative data handling. Our negative data handling approach does not assume non-reported PTM sites as negative data points given that much of the experimental data does not reflect all possible biological conditions (spatial, temporal, etc.) that can exist in nature. We will add a section to the Results (Performance evaluation and comparison to other models) and Discussion (6th paragraph) section to address this.
