# Supplementary material for: CLASPP: A unified model for predicting post-translational modifications
Source: PLoS Comput Biol. 2026 Aug 14;22(8):e1014616. doi: 10.1371/journal.pcbi.1014616 (PMC13475974; doi:10.1371/journal.pcbi.1014616)
Supplement: Supplementary file 5 [file pcbi.1014616.s011.pdf]

Comment 1

The strategy of dividing a single PTM type into multiple sub-clusters and training them separately is an interesting and novel idea. However, this approach effectively treats instances belonging to other sub-clusters of the same PTM type as negative examples during training. While such a strategy may be beneficial for highly abundant PTM types with diverse sequence patterns, its utility for PTM types with limited data is less clear, since further partitioning already scarce positive samples may reduce the amount of information available for learning.

More importantly, the central motivation of CLASPP is to address the severe data imbalance among PTM types. In this context, it would be helpful for the authors to explain how subdividing PTM types into multiple sub-clusters is expected to alleviate, rather than exacerbate, the data scarcity problem for underrepresented PTM categories.

Comment 2

The results presented in Supplementary Figure S4e appear to indicate that, with the exception of ST_Phosphorylation, CLASPP underperforms PTMGPT2 for many PTM types. This observation seems consistent with the concern raised above regarding the effectiveness of the sub-clustering strategy for PTM types with limited training data.

Therefore, I find it difficult to fully support the claim that CLASPP represents a state-of-the-art PTM prediction model. This concern becomes more significant when considering model complexity: existing methods such as DeepMVP and MusiteDeep contain only a few million trainable parameters, whereas CLASPP contains approximately 1.4 billion parameters. Given the substantially larger model size, the observed performance improvements appear limited and, for many PTM types, are not evident. The authors should provide a more balanced discussion of the trade-off between model complexity and predictive performance.

Comment 3

The description of data sampling and class balancing during training and evaluation remains insufficient. Additional details are needed regarding how positive and negative samples were selected and balanced throughout the workflow.

For example, in the confusion matrix for R_Methylation shown in Supplementary Figure S4e, there are 693 positive samples but only 100 negative samples. Such a class distribution appears highly unusual for a binary classification benchmark and may substantially affect the reported performance metrics. The authors should explain the rationale behind this sampling strategy and discuss its implications for model evaluation.

(Minor) Comment 4

In the previous revision, the authors explained that the increase in sample counts between Stages 3 and 4 of the curation pipeline was caused by the separate handling of multi-label PTM sites during Stages 2 and 4. While this clarification was helpful, the explanation should also be incorporated into the manuscript itself, either in the main text or in the caption of Supplementary Figure S2, so that readers can correctly interpret the reported sample counts.

(Minor) Comment 5

A few minor inconsistencies and typographical errors should be corrected.

First, the confusion matrix for K_Ubiquitination appears to be missing from Supplementary Figure S4e. Second, the F1 score for Y_Phosphorylation reported in the main text appears to be incorrect; according to Figure S4a, the F1 score for Y_Phosphorylation is 0.84.
