# Supplementary material for: CLASPP: A unified model for predicting post-translational modifications
Source: PLoS Comput Biol. 2026 Aug 14;22(8):e1014616. doi: 10.1371/journal.pcbi.1014616 (PMC13475974; doi:10.1371/journal.pcbi.1014616)
Supplement: Supplementary file 6 [file pcbi.1014616.s012.pdf]

Reviewer 1 Comment 1

The strategy of dividing a single PTM type into multiple sub-clusters and training them separately is an interesting and novel idea. However, this approach effectively treats instances belonging to other sub-clusters of the same PTM type as negative examples during training. While such a strategy may be beneficial for highly abundant PTM types with diverse sequence patterns, its utility for PTM types with limited data is less clear, since further partitioning already scarce positive samples may reduce the amount of information available for learning.

More importantly, the central motivation of CLASPP is to address the severe data imbalance among PTM types. In this context, it would be helpful for the authors to explain how subdividing PTM types into multiple sub-clusters is expected to alleviate, rather than exacerbate, the data scarcity problem for underrepresented PTM categories.

Response: We thank the reviewer for raising this important point. We agree that sub-clustering can be counterproductive for PTM types with limited data because it may further reduce the number of positive examples available within each subclass. Therefore, sub-clustering was not intended as a universal strategy for all PTM types. Rather, it was applied only to PTM categories that were sufficiently abundant and heterogeneous to support subdivision. Empirical hold-out, clustering quality, and out-of-distribution performance were then used to determine whether the resulting subclasses were retained in the final model. This is now clarified in the revised methods (Line 614).

Reviewer 1 Comment 2

The results presented in Supplementary Figure S4e appear to indicate that, with the exception of ST_Phosphorylation, CLASPP underperforms PTMGPT2 for many PTM types. This observation seems consistent with the concern raised above regarding the effectiveness of the sub-clustering strategy for PTM types with limited training data. Therefore, I find it difficult to fully support the claim that CLASPP represents a state-of-the-art PTM prediction model. This concern becomes more significant when considering model complexity: existing methods such as DeepMVP and MusiteDeep contain only a few million trainable parameters, whereas CLASPP contains approximately 1.4 billion parameters. Given the substantially larger model size, the observed performance improvements appear limited and, for many PTM types, are not evident. The authors should provide a more balanced discussion of the trade-off between model complexity and predictive performance.

Response: We thank the reviewer for this comment. To address this, we provide a size-capability plot for the aggregate and average member model size (Sfig. 4f). This allows for

better transparency on model complexity and performance. The results and discussion have been modified to reflect these changes.

Reviewer 1 Comment 3

The description of data sampling and class balancing during training and evaluation remains insufficient. Additional details are needed regarding how positive and negative samples were selected and balanced throughout the workflow.

For example, in the confusion matrix for R_Methylation shown in Supplementary Figure S4e, there are 693 positive samples but only 100 negative samples. Such a class distribution appears highly unusual for a binary classification benchmark and may substantially affect the reported performance metrics. The authors should explain the rationale behind this sampling strategy and discuss its implications for model evaluation

Response: We agree that changes in the positive/negative sample ratio can influence absolute performance metrics. The negative samples for R-methylation were not manually selected. Rather, they resulted from the same predefined PTM-type-stratified sampling and curation procedure applied consistently across all benchmarks. Because this strategy was designed to improve uniform representation across PTM types, it also affected the positive/negative ratio and the composition of the negative set. Instead of allowing individual protein sequence length and residue composition to determine benchmark class ratios, we used PTM-type-stratified sampling to define positive and negative sets independently of the composition of the contributing proteins. We have revised the Discussion to acknowledge the implications of class-ratio-dependent evaluation.

Reviewer 1 (Minor) Comment 4

In the previous revision, the authors explained that the increase in sample counts between Stages 3 and 4 of the curation pipeline was caused by the separate handling of multi-label PTM sites during Stages 2 and 4. While this clarification was helpful, the explanation should also be incorporated into the manuscript itself, either in the main text or in the caption of Supplementary Figure S2, so that readers can correctly interpret the reported sample counts.

Response: We have modified the curation overview to include additional details.

Reviewer 1 (Minor) Comment 5

A few minor inconsistencies and typographical errors should be corrected. First, the confusion matrix for K_Ubiquitination appears to be missing from Supplementary Figure S4e. Second, the F1 score for Y_Phosphorylation reported in the main text appears to be incorrect; according to Figure S4a, the F1 score for Y_Phosphorylation is 0.84

Response: These issues have been corrected in the revised version.
