# Supplementary material for: CLASPP: A unified model for predicting post-translational modifications
Source: PLoS Comput Biol. 2026 Aug 14;22(8):e1014616. doi: 10.1371/journal.pcbi.1014616 (PMC13475974; doi:10.1371/journal.pcbi.1014616)
Supplement: Peer review history

# Peer review: Decision Letter 0

**DOI:** 10.1371/journal.pcbi.1014616.r001

**Authors:** Arne Elofsson, Arli Aditya Parikesit

17 Nov 2025

CLASPP: A unified model for predicting post-translational modifications

PLOS Computational Biology

Dear Dr. Kannan,

Thank you for submitting your manuscript to PLOS Computational Biology. After careful consideration, we feel that it has merit but does not fully meet PLOS Computational Biology's publication criteria as it currently stands. Therefore, we invite you to submit a revised version of the manuscript that addresses the points raised during the review process.

Please submit your revised manuscript by Jan 17 2026 11:59PM. If you will need more time than this to complete your revisions, please reply to this message or contact the journal office at ploscompbiol@plos.org. When you're ready to submit your revision, log on to https://www.editorialmanager.com/pcompbiol/ and select the 'Submissions Needing Revision' folder to locate your manuscript file.

Please include the following items when submitting your revised manuscript:

* A rebuttal letter that responds to each point raised by the editor and reviewer(s). You should upload this letter as a separate file labeled 'Response to Reviewers'. This file does not need to include responses to formatting updates and technical items listed in the 'Journal Requirements' section below.

* A marked-up copy of your manuscript that highlights changes made to the original version. You should upload this as a separate file labeled 'Revised Manuscript with Track Changes'.

* An unmarked version of your revised paper without tracked changes. You should upload this as a separate file labeled 'Manuscript'.

If you would like to make changes to your financial disclosure, competing interests statement, or data availability statement, please make these updates within the submission form at the time of resubmission. Guidelines for resubmitting your figure files are available below the reviewer comments at the end of this letter

We look forward to receiving your revised manuscript.

Kind regards,

Arli Aditya Parikesit, PhD

Academic Editor

PLOS Computational Biology

Arne Elofsson

Section Editor

PLOS Computational Biology

**Additional Editor Comments :**

The reviewers have concluded their reports. Please kindly provide a letter of reply to address reviewers' concerns. Keep in mind for the validation of your pipeline, and comprehensive benchmarking of your pipeline as well.

**Journal Requirements:**

1) We ask that a manuscript source file is provided at Revision. Please upload your manuscript file as a .doc, .docx, .rtf or .tex. If you are providing a .tex file, please upload it under the item type u2018LaTeX Source Fileu2019 and leave your .pdf version as the item type u2018Manuscriptu2019.

2) Some material included in your submission may be copyrighted. According to PLOSu2019s copyright policy, authors who use figures or other material (e.g., graphics, clipart, maps) from another author or copyright holder must demonstrate or obtain permission to publish this material under the Creative Commons Attribution 4.0 International (CC BY 4.0) License used by PLOS journals. Please closely review the details of PLOSu2019s copyright requirements here: PLOS Licenses and Copyright. If you need to request permissions from a copyright holder, you may use PLOS's Copyright Content Permission form.

Please respond directly to this email and provide any known details concerning your material's license terms and permissions required for reuse, even if you have not yet obtained copyright permissions or are unsure of your material's copyright compatibility. Once you have responded and addressed all other outstanding technical requirements, you may resubmit your manuscript within Editorial Manager.

Potential Copyright Issues:

i) Figures 1, 5A, S1, S2B, S5A, and Striking Image. Please confirm whether you drew the images / clip-art within the figure panels by hand. If you did not draw the images, please provide (a) a link to the source of the images or icons and their license / terms of use; or (b) written permission from the copyright holder to publish the images or icons under our CC BY 4.0 license. Alternatively, you may replace the images with open source alternatives. See these open source resources you may use to replace images / clip-art:

- https://commons.wikimedia.org

- https://openclipart.org/.

3) Thank you for stating " Training datasets for both finetuning and contrastive learning are available here in this Zenodo repo(https://doi.org/10.5281/zenodo.16642476)." We noted that the data files are restricted to users with access. Please assure the datasets are publicly available.Please note that, though access restrictions are acceptable now, your entire minimal dataset will need to be made freely accessible if your manuscript is accepted for publication. This policy applies to all data except where public deposition would breach compliance with the protocol approved by your research ethics board.

4)  Your current Financial Disclosure states, "The author(s) received no specific funding for this work.".

However, your funding information on the submission form indicates receiving a fund. Please ensure that the funders and grant numbers match between the Financial Disclosure field and the Funding Information tab in your submission form. Note that the funders must be provided in the same order in both places as well.

Please amend your detailed Financial Disclosure statement. This is published with the article. It must therefore be completed in full sentences and contain the exact wording you wish to be published.

1) Please clarify all sources of financial support for your study. List the grants, grant numbers, and organizations that funded your study, including funding received from your institution. Please note that suppliers of material support, including research materials, should be recognized in the Acknowledgements section rather than in the Financial Disclosure

2) State the initials, alongside each funding source, of each author to receive each grant. For example: "This work was supported by the National Institutes of Health (####### to AM; ###### to CJ) and the National Science Foundation (###### to AM)."

3) State what role the funders took in the study. If the funders had no role in your study, please state: "The funders had no role in study design, data collection and analysis, decision to publish, or preparation of the manuscript."

4) If any authors received a salary from any of your funders, please state which authors and which funders..

5) Please modify your Competing Interests' statement in the online submission form and declare all competing interests beginning with the statement 'I have read the journal's policy and the authors of this manuscript have the following competing interests:'

Note: If the reviewer comments include a recommendation to cite specific previously published works, please review and evaluate these publications to determine whether they are relevant and should be cited. There is no requirement to cite these works unless the editor has indicated otherwise.

**Reviewers' comments:**

Reviewer's Responses to Questions

**Comments to the Authors:**

**Please note that two reviews are uploaded as attachments.**

Reviewer #1: review attached.

Reviewer #2: Detailed comments are provided in the attached review document.

Reviewer #3: 1. Use of Contrastive Learning Framework

Gravel et al. utilized a contrastive learning based, attention-driven stratified framework for predicting major PTMs.

2. Line 64 Correction

Replace labile with dynamic.

3. Choice of Embeddings (ESM2-3B vs. ESM-2-150M)

The manuscript does not justify why ESM2-3B contextualized embeddings were not used. Instead, the study relies on ESM-2-150M encoder embeddings. A clear rationale should be provided. Additionally, the dimensionality of the contextualized embeddings generated by the 150M model must be explicitly stated.

4. Exclusion of MCC Evaluation Metric

MCC (Matthews Correlation Coefficient) has been consistently used in prior PTM prediction studies, particularly due to its robustness with imbalanced datasets. The manuscript should explain why MCC was not included as an evaluation metric.

5. Figure and Typographical Corrections

• Figure 3A: Change “Losss” to “Loss.”

• Line 381: Replace “Muti” with “Multi.”

6. Construction of the Negative Dataset

The method used to construct the negative dataset is unclear. For example, if specific serine/threonine sites in a protein are annotated as phosphorylated, are the remaining non-annotated S/T sites in the same protein treated as negative? This is a standard requirement in PTM prediction. If this procedure was not followed, the manuscript cannot be considered acceptable. A detailed explanation of the negative data generation process is required.

7. Feature Vector Generation via ESM-2-150M

The manuscript should clearly describe how each 21-mer sequence was converted into feature vectors using the ESM-2-150M encoder, including the embedding structure. Additionally, the application of contrastive learning to these feature vectors needs to be explained step by step.

8. GitHub Repository

All PTM-related data (positive and negatives) should be provided in the following format:

Protein ID | PTM Site | 21-mer Peptide | Label | PTM Type

Example:

P12KYKU | 21 | ..RSLNKSTUT.. | Negative | Phosphorylation

Currently, no such data is available in the GitHub repository. You must provide all datasets used in the analysis in this exact format to ensure reproducibility and enable further study.

**********

**Have the authors made all data and (if applicable) computational code underlying the findings in their manuscript fully available?**

The PLOS Data policy requires authors to make all data and code underlying the findings described in their manuscript fully available without restriction, with rare exception (please refer to the Data Availability Statement in the manuscript PDF file). The data and code should be provided as part of the manuscript or its supporting information, or deposited to a public repository. For example, in addition to summary statistics, the data points behind means, medians and variance measures should be available. If there are restrictions on publicly sharing data or code —e.g. participant privacy or use of data from a third party—those must be specified.

Reviewer #1: **No:**  Code and model parameters were available, but training and test datasets were not accessible.

Reviewer #2: Yes

Reviewer #3: Yes

**********

PLOS authors have the option to publish the peer review history of their article (what does this mean?). If published, this will include your full peer review and any attached files.

If you choose “no”, your identity will remain anonymous but your review may still be made public.

**Do you want your identity to be public for this peer review?**  For information about this choice, including consent withdrawal, please see our Privacy Policy.

Reviewer #1: No

Reviewer #2: No

Reviewer #3: No

[NOTE: If reviewer comments were submitted as an attachment file, they will be attached to this email and accessible via the submission site. Please log into your account, locate the manuscript record, and check for the action link "View Attachments". If this link does not appear, there are no attachment files.]

**Figure resubmission:**

**Reproducibility:**

?>

Attachment Submitted filename: Review_CLASPP_20251104.pdf

Attachment Submitted filename: Review-SA-CLASPP.pdf

# Author comment: Author response to Decision Letter 1

**DOI:** 10.1371/journal.pcbi.1014616.r002

10 Feb 2026

Attachment Submitted filename: claspp_rev_com.pdf

# Peer review: Decision Letter 1

**DOI:** 10.1371/journal.pcbi.1014616.r003

**Authors:** Arne Elofsson, Arli Aditya Parikesit

16 Apr 2026

CLASPP: A unified model for predicting post-translational modifications

PLOS Computational Biology

Dear Dr. Kannan,

Thank you for submitting your manuscript to PLOS Computational Biology. After careful consideration, we feel that it has merit but does not fully meet PLOS Computational Biology's publication criteria as it currently stands. Therefore, we invite you to submit a revised version of the manuscript that addresses the points raised during the review process.

Please submit your revised manuscript by Jun 16 2026 11:59PM. If you will need more time than this to complete your revisions, please reply to this message or contact the journal office at ploscompbiol@plos.org. When you're ready to submit your revision, log on to https://www.editorialmanager.com/pcompbiol/ and select the 'Submissions Needing Revision' folder to locate your manuscript file.

Please include the following items when submitting your revised manuscript:

* A letter that responds to each point raised by the editor and reviewer(s). You should upload this letter as a separate file labeled 'Response to Reviewers'. This file does not need to include responses to formatting updates and technical items listed in the 'Journal Requirements' section below.

* A marked-up copy of your manuscript that highlights changes made to the original version. You should upload this as a separate file labeled 'Revised Manuscript with Track Changes'.

* An unmarked version of your revised paper without tracked changes. You should upload this as a separate file labeled 'Manuscript'.

If you would like to make changes to your financial disclosure, competing interests statement, or data availability statement, please make these updates within the submission form at the time of resubmission. Guidelines for resubmitting your figure files are available below the reviewer comments at the end of this letter

We look forward to receiving your revised manuscript.

Kind regards,

Arli Aditya Parikesit, PhD

Academic Editor

PLOS Computational Biology

Arne Elofsson

Section Editor

PLOS Computational Biology

**Reviewers' comments:**

Reviewer's Responses to Questions

**Comments to the Authors:**

**Please note here if the review is uploaded as an attachment.**

Reviewer #1: Comment 1: In Supplementary Figure 2E, several PTM types (e.g., Glutathionylation, S-palmitoylation, and Hydroxylation) show higher counts in Stage 4 compared to Stage 3. Given that Stage 4 clustering is performed on the dataset already filtered in Stage 3, the number of entries cannot increase. The authors should clarify this discrepancy or revise the figure accordingly.

Comment 2: While limiting the number of positive samples to 2,000 helps balance the dataset, it may reduce the diversity of training examples from larger clusters. It would be helpful if the authors clarify how data diversity was maintained during training - for example, whether different subsets were resampled across epochs or runs, or whether a cross-validation strategy was used - to ensure that model performance is not dependent on a fixed subset of the data.

Comment 3: In Supplementary Figure 2C, the within-cluster distance is typically expected to decrease as the number of clusters increases, due to reduced intra-cluster variance. The authors should provide a more detailed explanation of the trends observed in this figure.

Comment 4: Since CLASPP defines three different types of negative samples, additional clarification is required regarding how the negative datasets were constructed and used for performance comparison with benchmark models (shown in Figure 4).

Comment 5: While Supplementary Figure 4A presents model performance across PTM types, interpretation of these results is not entirely straightforward. Specifically, some PTM types appear to exhibit simultaneously high false positive rates and high precision, which is counterintuitive and makes it difficult to assess the model's predictive behavior. To improve transparency, the authors should include confusion matrices for these PTM types to clarify how these metrics were derived.

Comment 6: The authors are encouraged to report the total number of trainable parameters, as this information would help readers better assess model complexity and enable fair comparison with existing methods.

Reviewer #3: Upon reviewing the negative dataset in benchmark_test_HUMAN_hd3_Acetylation(AM)-25_05_11.csv, it is clear that the current methodology deviates from the rigorous standards required for PTM prediction.

The current sampling (using Hamming distance and random selection) does not reflect biological reality. In a rigorous PTM study, if a protein contains 10 Lysine (K) residues and only two (e.g., K24 and K160) are annotated as positive, the remaining eight K residues must be treated as the negative data. This is the only way to ensure the model learns to distinguish a true modification site from a biologically similar but unmodified site.

To meet the standard required for high-impact publication, please redo all experiments using this 'non-annotated site' approach. Specifically, generate a fourth category of negative data based strictly on this logic and update all performance metrics. This revision is mandatory for the next iteration of the manuscript. As well as release all the dataset relevant to it.

**********

**Have the authors made all data and (if applicable) computational code underlying the findings in their manuscript fully available?**

The PLOS Data policy requires authors to make all data and code underlying the findings described in their manuscript fully available without restriction, with rare exception (please refer to the Data Availability Statement in the manuscript PDF file). The data and code should be provided as part of the manuscript or its supporting information, or deposited to a public repository. For example, in addition to summary statistics, the data points behind means, medians and variance measures should be available. If there are restrictions on publicly sharing data or code —e.g. participant privacy or use of data from a third party—those must be specified.

Reviewer #1: Yes

Reviewer #3: Yes

**********

PLOS authors have the option to publish the peer review history of their article (what does this mean?). If published, this will include your full peer review and any attached files.

If you choose “no”, your identity will remain anonymous but your review may still be made public.

**Do you want your identity to be public for this peer review?**  For information about this choice, including consent withdrawal, please see our Privacy Policy.

Reviewer #1: No

Reviewer #3: No

[NOTE: If reviewer comments were submitted as an attachment file, they will be attached to this email and accessible via the submission site. Please log into your account, locate the manuscript record, and check for the action link "View Attachments". If this link does not appear, there are no attachment files.]

**Figure resubmission:**

**Reproducibility:**

?>

# Author comment: Author response to Decision Letter 2

**DOI:** 10.1371/journal.pcbi.1014616.r004

4 Jun 2026

Attachment Submitted filename: claspp_reviewer_comments.pdf

# Peer review: Decision Letter 2

**DOI:** 10.1371/journal.pcbi.1014616.r005

**Authors:** Arne Elofsson, Arli Aditya Parikesit

29 Jun 2026

PCOMPBIOL-D-25-01997R2

CLASPP: A unified model for predicting post-translational modifications

PLOS Computational Biology

Dear Dr. Kannan,

Thank you for submitting your manuscript to PLOS Computational Biology. After careful consideration, we feel that it has merit but does not fully meet PLOS Computational Biology's publication criteria as it currently stands. Therefore, we invite you to submit a revised version of the manuscript that addresses the points raised during the review process.

Please submit your revised manuscript by Aug 29 2026 11:59PM. If you will need more time than this to complete your revisions, please reply to this message or contact the journal office at ploscompbiol@plos.org. When you're ready to submit your revision, log on to https://www.editorialmanager.com/pcompbiol/ and select the 'Submissions Needing Revision' folder to locate your manuscript file.

Please include the following items when submitting your revised manuscript:

* A letter that responds to each point raised by the editor and reviewer(s). You should upload this letter as a separate file labeled 'Response to Reviewers'. This file does not need to include responses to formatting updates and technical items listed in the 'Journal Requirements' section below.

* A marked-up copy of your manuscript that highlights changes made to the original version. You should upload this as a separate file labeled 'Revised Manuscript with Track Changes'.

* An unmarked version of your revised paper without tracked changes. You should upload this as a separate file labeled 'Manuscript'.

If you would like to make changes to your financial disclosure, competing interests statement, or data availability statement, please make these updates within the submission form at the time of resubmission. Guidelines for resubmitting your figure files are available below the reviewer comments at the end of this letter.

As the corresponding author, your ORCID iD is verified in the submission system and will appear in the published article. PLOS supports the use of ORCID, and we encourage all coauthors to register for an ORCID iD and use it as well. Please encourage your coauthors to verify their ORCID iD within the submission system before final acceptance, as unverified ORCID iDs will not appear in the published article. Only the individual author can complete the verification step; PLOS staff cannot verify ORCID iDs on behalf of authors.

We look forward to receiving your revised manuscript.

Kind regards,

Arli Aditya Parikesit, PhD

Academic Editor

PLOS Computational Biology

Arne Elofsson

Section Editor

PLOS Computational Biology

**Additional Editor Comments (if provided):**

Minor revisions are still needed. Kindly check If the reviewer is providing the commented manuscript or attachment for follow ups

**Journal Requirements:**

If the reviewer comments include a recommendation to cite specific previously published works, please review and evaluate these publications to determine whether they are relevant and should be cited. There is no requirement to cite these works unless the editor has indicated otherwise.

1) Please upload all main figures as separate Figure files in .tif or .eps format. For more information about how to convert and format your figure files please see our guidelines:

https://journals.plos.org/ploscompbiol/s/figures

2) Please upload a copy of Figures FIG 1A-C, 2 A-C, 3A-C, 4A-C, 5A-C, and 6 which you refer to in your text on pages 7, 9, 14, 18, 22, and 24. Or, if the figure is no longer to be included as part of the submission please remove all reference to it within the text.

**Reviewers' comments:**

Reviewer's Responses to Questions

**Comments to the Authors:**

**Please note here if the review is uploaded as an attachment.**

Reviewer #1: Review attached.

Reviewer #3: Good work.

**********

**Have the authors made all data and (if applicable) computational code underlying the findings in their manuscript fully available?**

The PLOS Data policy requires authors to make all data and code underlying the findings described in their manuscript fully available without restriction, with rare exception (please refer to the Data Availability Statement in the manuscript PDF file). The data and code should be provided as part of the manuscript or its supporting information, or deposited to a public repository. For example, in addition to summary statistics, the data points behind means, medians and variance measures should be available. If there are restrictions on publicly sharing data or code —e.g. participant privacy or use of data from a third party—those must be specified.

Reviewer #1: Yes

Reviewer #3: Yes

**********

PLOS authors have the option to publish the peer review history of their article (what does this mean?). If published, this will include your full peer review and any attached files.

If you choose “no”, your identity will remain anonymous but your review may still be made public.

**Do you want your identity to be public for this peer review?**  For information about this choice, including consent withdrawal, please see our Privacy Policy.

Reviewer #1: No

Reviewer #3: No

[NOTE: If reviewer comments were submitted as an attachment file, they will be attached to this email and accessible via the submission site. Please log into your account, locate the manuscript record, and check for the action link "View Attachments". If this link does not appear, there are no attachment files.]

**Figure resubmission:**

**Reproducibility:**

To enhance the reproducibility of your results, we recommend that authors of applicable studies deposit laboratory protocols in protocols.io, where a protocol can be assigned its own identifier (DOI) such that it can be cited independently in the future. Additionally, PLOS ONE offers an option to publish peer-reviewed clinical study protocols. Read more information on sharing protocols at https://plos.org/protocols?utm_medium=editorial-email&utm_source=authorletters&utm_campaign=protocols

Attachment Submitted filename: Revision2_ReviewComment.pdf

# Author comment: Author response to Decision Letter 3

**DOI:** 10.1371/journal.pcbi.1014616.r006

17 Jul 2026

Attachment Submitted filename: Response_reviewer.pdf

# Editor report: Decision Letter 3

**DOI:** 10.1371/journal.pcbi.1014616.r007

**Authors:** Arne Elofsson, Arli Aditya Parikesit

22 Jul 2026

Dear Dr. Kannan,

We are pleased to inform you that your manuscript 'CLASPP: A unified model for predicting post-translational modifications' has been provisionally accepted for publication in PLOS Computational Biology.

Before your manuscript can be formally accepted you will need to complete some formatting changes, which you will receive in a follow up email. A member of our team will be in touch with a set of requests.

Please note that your manuscript will not be scheduled for publication until you have made the required changes, so a swift response is appreciated.

IMPORTANT: The editorial review process is now complete. PLOS will only permit corrections to spelling, formatting or significant scientific errors from this point onwards. Requests for major changes, or any which affect the scientific understanding of your work, will cause delays to the publication date of your manuscript.

Should you, your institution's press office or the journal office choose to press release your paper, you will automatically be opted out of early publication. We ask that you notify us now if you or your institution is planning to press release the article. All press must be co-ordinated with PLOS.

Thank you again for supporting Open Access publishing; we are looking forward to publishing your work in PLOS Computational Biology.

Best regards,

Arli Aditya Parikesit, PhD

Academic Editor

PLOS Computational Biology

Arne Elofsson

Section Editor

PLOS Computational Biology

***********************************************************

The authors have addressed reviewer's comments accordingly. Therefore, I decided to accept this manuscript for publication.

# Editor report: Acceptance letter

**DOI:** 10.1371/journal.pcbi.1014616.r008

**Authors:** Arne Elofsson, Arli Aditya Parikesit

PCOMPBIOL-D-25-01997R3

CLASPP: A unified model for predicting post-translational modifications

Dear Dr Kannan,

I am pleased to inform you that your manuscript has been formally accepted for publication in PLOS Computational Biology. Your manuscript is now with our production department and you will be notified of the publication date in due course.

The corresponding author will soon be receiving a typeset proof for review, to ensure errors have not been introduced during production. Please review the PDF proof of your manuscript carefully, as this is the last chance to correct any errors. Please note that major changes, or those which affect the scientific understanding of the work, will likely cause delays to the publication date of your manuscript.

Soon after your final files are uploaded, unless you have opted out, the early version of your manuscript will be published online. The date of the early version will be your article's publication date. The final article will be published to the same URL, and all versions of the paper will be accessible to readers.

For Research, Software, and Methods articles, you will receive an invoice from PLOS for your publication fee after your manuscript has reached the completed accept phase. If you receive an email requesting payment before acceptance or for any other service, this may be a phishing scheme. Learn how to identify phishing emails and protect your accounts at https://explore.plos.org/phishing.

Thank you again for supporting PLOS Computational Biology and open-access publishing. We are looking forward to publishing your work!

With kind regards,

Janani Seenivasan

PLOS Computational Biology | Carlyle House, Carlyle Road, Cambridge CB4 3DN | United Kingdom ploscompbiol@plos.org | Phone +44 (0) 1223-442824 | ploscompbiol.org | @PLOSCompBiol
